# Metabolomic Analysis of Lytic KSHV Infection: Induced Host Nucleotide Metabolism Is Required for Infectious Virus Production

**DOI:** 10.3390/v18080877

**Published:** 2026-08-11

**Authors:** Fatima Hisam, Emma A. Winn, Spandan Mukherjee, Savannah E. Price, Yennifer A. Gaspar, Claire Wang, Hamid R. Baniasadi, Tracie Delgado, Erica L. Sanchez

**Affiliations:** 1 Department of Biological Sciences, The University of Texas at Dallas, Richardson, TX 75080, USA; fatima.hisam@utdallas.edu (F.H.); emma.winn@utdallas.edu (E.A.W.); spandan.mukherjee@utdallas.edu (S.M.); savannah.price@utdallas.edu (S.E.P.); claire.wang@utdallas.edu (C.W.); 2 Department of Biology, Seattle Pacific University, Seattle, WA 98119, USA; yenni.gaspar21@gmail.com (Y.A.G.); delgadot@spu.edu (T.D.); 3 Department of Biochemistry, UT Southwestern Medical Center, Dallas, TX 75390, USA; hamid.baniasadi@utsouthwestern.edu

**Keywords:** Kaposi’s Sarcoma Herpesvirus (KSHV), Kaposi’s Sarcoma (KS), metabolomics, methotrexate (MTX), nucleotide metabolism, lytic replication

## Abstract

Kaposi’s Sarcoma Herpesvirus (KSHV) is the etiological agent of Kaposi’s Sarcoma (KS), which induces metabolic stress in infected host cells. KSHV reprograms host metabolic pathways for efficient viral replication and infectious virion production. Here, we report a time-course global metabolomics study conducted in iSLK.BAC16 cells to compare latent and lytic KSHV infection. Our data show that amino acid, central carbon, and nucleotide metabolic pathways are highly dysregulated upon reactivation. During lytic KSHV infection, pathway enrichment analysis identifies purine and pyrimidine metabolism as the top two most significantly impacted and dysregulated pathways. Further experiments have shown that nucleotide metabolism is required during lytic KSHV infection to produce maximal infectious virus. Treatment with the FDA-approved drug, methotrexate (MTX), a folate antagonist that decreases nucleotide metabolism by reducing tetrahydrofolate cofactors, significantly reduced KSHV copy number and late lytic viral gene expression upon reactivation compared to controls. Additionally, titers of cell-free supernatants from MTX-treated lytic samples showed a significant reduction in infectious virion production. Furthermore, MTX significantly decreased the viral titer of murine herpesvirus 68 (MHV-68), a model virus to study gammaherpesvirus. Overall, our study demonstrates that metabolic inhibition during lytic gammaherpesvirus infection decreases productive infection and hence serves as a potential therapeutic antiviral target.

## 1. Introduction

Kaposi’s Sarcoma-associated Herpesvirus (KSHV), an oncogenic gammaherpesvirus, is the causative agent of Kaposi’s Sarcoma (KS) [1,2]. KS is a soft-tissue cancer that presents as red-purple lesions on the skin and mucous membranes. KSHV is also involved in other human malignancies, including primary effusion lymphoma (PEL), multicentric Castleman’s disease (MCD), and KSHV inflammatory cytokine syndrome (KICS) [1,3]. KSHV causes cancer in immunosuppressed individuals and is the most common cancer in AIDS patients across the globe [3,4].

Like all herpesviruses, KSHV is a double-stranded DNA virus [5] enclosed in a protein capsid and then in a lipid bilayer [6]. KSHV undergoes two distinct viral phases: latent and lytic infection. Most cells in KS lesions are latently infected, with approximately 5% undergoing lytic replication. The latent phase is defined by limited viral gene expression, including the expression of the latency-associated nuclear antigen (LANA). This stage results in no new virion production, and the host cell survives and proliferates [7]. The lytic viral phase expresses all the genes in a temporal fashion, which includes immediate-early and early viral gene expression, viral genome replication, late viral gene expression, virion assembly, and egress from the cell. Representative viral genes investigated in this study are the latent gene LANA; the early lytic viral genes Replication and Transcriptional Activator (RTA, ORF50) and DNA polymerase (ORF59); and the late lytic viral gene glycoprotein K8.1 [8].

Viruses are obligate parasites that depend on the host cell for their growth and survival. Host cell metabolism is one of the major cellular systems manipulated during KSHV infection. KSHV, like other herpesviruses, commandeers host cell metabolism for energy production and raw material synthesis for viral replication and production [9,10]. However, the virus–host cell interactions and many of the underlying mechanisms of these viral manipulations of cellular metabolic pathways remain unclear.

The field of metabolomics has emerged as a key discipline in the wake of genomics, transcriptomics, and proteomics [11]. Studies have been performed to quantify the levels of metabolic compounds (energy molecules and biochemical building blocks) during viral infection in host cells by employing an advanced measurement technique, liquid chromatography–mass spectrometry (LC-MS) [12]. Previous studies have shown that viral infections—such as Human Cytomegalovirus (HCMV), Epstein–Barr virus (EBV), Hepatitis C (HCV), Human Immunodeficiency Virus (HIV), Dengue, and Herpes Simplex Virus (HSV-1)—lead to dramatic increases in the levels of many metabolites. These increases substantially exceed those associated with normal transitions of cells between resting and growing states. In several studies, differential metabolite abundances induced by viral infection coincide with an increase in host cell expression of enzymes that drive metabolite synthesis [13,14,15,16,17,18].

Mass spectrometry-based metabolomic approaches confirmed the divergent metabolic profile of two related herpesviruses, HCMV and HSV-1 [16]. These viruses require different types of metabolites based on the duration of the viral replicative cycle. HSV-1 has an average replication cycle of 24 h and requires rapid accumulation of nucleotides, which is marked by a significantly increased demand for DNA synthesis. On the other hand, HCMV accumulates nucleotides over the viral replication cycle of ~96 h, altering the glycolytic metabolites used in the production of fatty acid synthesis and inducing the production of new lipid membranes [16].

Previously, both gas chromatography–mass spectrometry (GC-MS) and/or liquid chromatography–mass spectrometry (LC-MS) methods were performed on latent KSHV-infected endothelial cells compared to mock-infected controls, which demonstrated that significant metabolic pathways were altered by latent infection, similar to those altered in cancer cells [19]. Latent KSHV infection increases amino acid metabolism, including polyamine metabolism (putrescine, spermidine, and spermine), at 48 and 96 h post-infection [19]. The latent infection also alters glycolysis and induces the Warburg Effect in the infected cells, resulting in decreased glycolytic flux to oxidative phosphorylation and increased lactate production, a key hallmark of cancer [20,21]. KSHV latent infection also requires lipogenesis and increased lipid droplet formation for lipid storage, which is necessary for virion assembly [19,21]. The requirement of cellular metabolism for KSHV latent infection was further correlated with proteome and phosphoproteome analysis [22]. Untargeted metabolomics of KSHV latent infection versus mock determined the metabolic changes induced after four hours of latent infection, including the urea cycle, polyamine synthesis, dimethylarginine synthesis, and de novo pyrimidine synthesis [23]. A recent KSHV study explored mechanistic biochemistry and metabolic pathway manipulation, which focused on metabolic rewiring that supports latent cell proliferation. The study demonstrated that KSHV hijacks host nucleotide synthesis and glycolysis via carbamoyl-phosphate synthetase 2, aspartate transcarbamoylase, and dihydroorotase (CAD), and via RelA, thereby promoting viral persistence and inducing proliferation [24]. Overall, these previous studies highlighted metabolic pathways implicated in KSHV infection; however, further exploration of host cell metabolic alterations during KSHV lytic replication is necessary.

Recently, metabolomic and lipidomic analysis performed on murine herpesvirus 68 (MHV-68)—a mouse gammaherpesvirus which shares significant homology to human gammaherpesvirus KSHV and EBV—induced alterations in key metabolic pathways, including glycolysis, glutaminolysis, lipid metabolism, and nucleotide metabolism [25]. Interestingly, nucleotide metabolism was the most highly increased metabolic pathway in the lytic MHV-68 metabolomics. Furthermore, inhibition of glycolysis and lipogenesis through pharmacological drugs and inhibition of glutaminolysis through starvation decreased infectious MHV-68 virus production. These results suggest that lytic gammaherpesvirus infection is highly dependent on the alteration of key metabolic pathways in favor of infectious virion production. However, this has yet to be evaluated in a lytic model of KSHV infection.

Here, we report that KSHV viral replication is dependent on host nucleotide metabolism. The experiments were conducted using the iSLK.BAC16 cell line, which stably maintains the recombinant KSHV-BAC16 encoding constitutive expression of GFP [26]. These cells encode the viral Replication and Transcription Activator (RTA) transgene and are doxycycline (Dox) and sodium butyrate (NaB) inducible, which triggers the transition from latent to lytic infection [26]. LC-MS/MS analysis of lytic KSHV infection in iSLK.BAC16 cells, compared with latent infection, revealed alterations in host metabolic pathways, including amino acid metabolism, central carbon pathways, and nucleotide metabolism. Metabolic pathway enrichment analysis revealed the top two most significantly impacted and dysregulated pathways to be purine and pyrimidine metabolism. Our LC-MS/MS data revealed increased abundances of key purine/pyrimidine-triphosphate species during lytic infection, suggesting an amplified shift in nucleotide biosynthesis, potentially to support the production of more viral genes and transcripts to sustain infection. Inhibition of pyrimidine and purine synthesis via the FDA-approved drug methotrexate (MTX) significantly reduced extracellular viral titers of KSHV and MHV-68. MTX, a competitive inhibitor of host dihydrofolate reductase (DHFR), is a folate antagonist that is used as a chemotherapeutic agent and immunosuppressive drug inhibitor [27,28,29]. MTX functions by decreasing tetrahydrofolate (THF), which acts as a substrate for subsequent metabolic steps [30,31]. Additionally, MTX treatment of iSLK.BAC16 cells demonstrated a significant reduction in viral genome copy number and late lytic viral mRNA expression.

Our global metabolomic analysis revealed changes in key metabolites of central carbon pathways, amino acid metabolism, and nucleotide metabolism during the infection time course after reactivation. Furthermore, we demonstrate that both KSHV and MHV-68 lytic infections require functional nucleotide metabolism for maximal viral infection. Understanding how KSHV lytic infection drives virus-induced global host cell metabolic changes and recapitulating the importance of altered host nucleotide metabolism during MHV-68 lytic infection can provide us with insights into new therapeutic mechanisms to manage KSHV-associated malignancies.

## 2. Materials and Methods

### 2.1. Cells and Reagents

iSLK.BAC16 cells [26] or iSLK cells [32] were maintained at 37 °C in 5% CO_2_ as monolayer cultures in Dulbecco’s modified Eagle medium (DMEM) (Corning, Corning, NY, USA, 10-013-CV) with L-glutamine (Corning, Corning, NY, USA, 25030-081) and sodium pyruvate, additionally supplemented with 10% fetal bovine serum (FBS) (Corning, Corning, NY, USA, 35-011-CV) and 1% L-glutamine. iSLK.BAC16 cell medium was selected with puromycin (10 mg/mL), G418 (95 mg/mL), and hygromycin B (Corning, Corning, NY, USA, 30-240-CR; 50 mg/mL). The iSLK cell medium was supplemented with puromycin (10 mg/mL) and G418 (95 mg/mL). All KSHV lytic experiments were carried out in DMEM with 10% FBS and 1% L-glutamine, without selective agents.

For the MHV-68 studies, NIH 3T3 cells (ATCC, Manassas, VA, USA, no. CRL-1658) or Vero cells (ATCC, Manassas, VA, USA, no. CCL-81) were cultured at 37 °C and 5% CO_2_ in DMEM containing high glucose, L-glutamine, sodium pyruvate (Genesee, El Cajon, CA, USA, no. 25-500), and 1% penicillin–streptomycin (Genesee, El Cajon, CA, USA, no. 25-512). Additionally, NIH 3T3 cells were supplemented with 10% newborn calf serum (Fisher, Hampton, NH, USA, #16010159), and Vero cells were supplemented with 10% fetal bovine serum (Genesee, El Cajon, CA, USA, no. 25-550).

### 2.2. Virus

The iSLK.BAC16 cell line stably maintains the KSHV genome. The cells were latently infected with recombinant KSHV.BAC16, which encodes constitutive expression of GFP. These cells also encode the viral Replication and Transcription Activator (RTA) transgene, which is doxycycline (1 μg/mL) (Dox) and sodium butyrate (1 μg/mL) (NaB) inducible; this triggers the transition in iSLK.BAC16 from latent to lytic infection. iSLK cells do not contain the KSHV genome and, therefore, are used as a control in our experiments.

### 2.3. Drug Treatment for KSHV Infection

Methotrexate (MTX) (ThermoScientific Chemicals, Tewksbury, MA, USA, J63075.MC) was prepared in DMSO and used at a final concentration of 125 nM in DMEM. iSLK.BAC16 cells were seeded on day 1 and reactivated (Dox and NaB) the next day. MTX was added at the time of reactivation. At 48 h post-treatment, the cell-free supernatant was collected and saved to perform virus titer experiments. The cells were harvested for qPCR analysis.

### 2.4. MHV-68 Infection, MTX Treatment, Viral Titer, and Cell Counts

MTX (Sigma, Burlington, MA, USA, no. 1414003) was dissolved in dimethyl sulfoxide (DMSO) and stored at −80 °C until use. NIH 3T3 cells were seeded at a density of 760,000 cells in 6 cm dishes. MHV-68 viral stocks (ATCC, Manassas, VA, USA no. VR-1465) were propagated via infection of NIH 3T3 cells or Vero cells at an MOI of 0.01–0.05 as previously described [25]. Approximately 4 h post-seeding, NIH 3T3 cells were mock- or MHV-68-infected (MOI = 0.1) in serum-free DMEM for 2 h and rocked every 20 min. After infection, the cell media was aspirated, replaced with 3 mL of fresh complete DMEM, and either vehicle (DMSO control) or 100 nM MTX was added. At 48 hpi, the viral supernatant was cleared by centrifugation (1000 rpm) and frozen at −80 °C for future plaque assay analysis. The cell pellets after centrifugation were combined with trypsinized cells from matching samples. Each sample—(1) Mock (DMSO control), (2) Mock (100 nM MTX), (3) MHV-68 (DMSO control), and (4) MHV-68 (100 nM MTX)—was centrifuged at 125× *g* for 5 min. The media was aspirated, and the cell pellet was resuspended in 500 µL complete DMEM. Total cell, live cell, and dead cell numbers were determined by a trypan blue exclusion assay using an automated Bio-Rad T-20 cell counter (Bio-Rad, Hercules, CA, USA, no. 1450102). Viral titers were quantified through traditional viral plaque assays and crystal violet staining as previously described [25]. Plaque-forming units per mL (pfu/mL) were calculated from duplicate wells using the equation: (dilution factor) × (1/0.2 mL) × (average pfu) = pfu/mL.

### 2.5. Quantitative Reverse Transcription–PCR (qRT-PCR)

All treated cells were washed once with phosphate-buffered saline (PBS) (Corning, 21-040-CV), and RNA was extracted following the manufacturer’s manual using the PureLink™ RNA Mini Kit (ThermoFisher Scientific, Waltham, MA, USA, 12183025). A two-step quantitative real-time reverse transcription PCR (RT-qPCR, Applied Biosystems Waltham, MA, USA) was used to measure mRNA expression levels of host metabolic genes and KSHV viral latent and lytic genes. Relative changes in gene expression were normalized using the delta threshold cycle (ΔCt) method to the abundance of host actin mRNA (housekeeping gene). The ΔCt values were then used to determine ΔΔCt when compared to the Dox-treated control group (iSLK.BAC16 cells treated with Dox and NaB).

### 2.6. Spinoculation Infection

All iSLK.BAC16 cell supernatants were collected post-experimental treatment (specifics described in the results section) and subjected to centrifugation for clearing debris at 300× *g* for 10 min. iSLK cells were seeded in 12-well plates at 70% confluency 24 h before induction. To determine the viral titer, 500 µL of supernatant from treated iSLK.BAC16 cells, supplemented with polybrene (10 µg/mL), was added to iSLK cells. Infection was enhanced by spinoculation at 300× *g*, 37 °C for 1 h and then incubated for 4 h at 37 °C with interval rocking plates every 30 min. Infectious supernatant media was replaced with fresh iSLK medium after 4 h. Titer plates were harvested after 48 h for flow cytometry experiments.

### 2.7. Flow Cytometry

iSLK cells were seeded for spinoculation as described above. After 48 h of infection, cells were washed once with PBS, trypsinized, collected, and pelleted by centrifugation at 300× *g* for 5 min. The supernatant was discarded, and the cell pellet was resuspended in 200 µL of FACS buffer (2% FBS in PBS). The cells were fixed in 4% paraformaldehyde for 5 min, washed with 2 mL PBS, and centrifuged for 5 min at 300× *g*. The supernatant was removed, and the pellet was resuspended in a 500 μL FACS buffer. For flow cytometry analysis, the samples were immediately analyzed using a flow cytometer equipped with filters suitable for detecting FITC. A BD Biosciences LSRFortessa SORP cytometer (Franklin Lakes, NJ, USA) was used for FACS data collection. During data collection, a minimum of 10,000 cells/events per sample was recorded to ensure statistical significance. In the data analysis phase, the acquired data were processed using FlowJo (v10.9.0). The population was first selected for live cells and then for singlets. The singlet cells were then analyzed for FITC to determine the number of KSHV-infected cells (GFP+).

### 2.8. Metabolite Extraction

iSLK.BAC16 cells were seeded in 60 mm dishes with five biological replicates per sample and harvested across a post-reactivation time course (24, 36, and 48 h). Cells were harvested for metabolomics, and the supernatant was saved for titering. Briefly, cells were washed with chilled PBS three times on dry ice and at −80 °C. Next, the metabolite extraction buffer, 80:20 methanol:water (*v*/*v*), was immediately added to quench cellular metabolism. The cells were scraped on dry ice, collected in microcentrifuge tubes, and centrifuged at high speed (14,000 rpm) for 15 min at 4 °C. The pellets were saved for protein quantification, and the supernatant with metabolites was centrifuged twice at high speed (14,000 rpm) for 15 min at 4 °C. Finally, the metabolite-containing solution was transferred to HPLC vials and transported to the UT Southwestern Medical Center Metabolomics Core Facility. Samples were stored at −80 °C until processed on a mass spectrometer. The core facility provided the metabolic profiling of the samples using targeted metabolomics.

### 2.9. LC-MS/MS Instrumentation System

Mass spectrometric analyses were performed on a Sciex “QTRAP 6500+” mass spectrometer (SCIEX, Marlborough, MA, USA) equipped with an ESI ion spray source. The ESI source was used in both positive- and negative-ion modes. The ion spray needle voltages used for MRM positive and negative polarity modes were set at 4800 V and −4500 V, respectively. The mass spectrometer was coupled to a Shimadzu HPLC (Nexera X2 LC-30AD, Shimadzu Corporation, Kyoto, Japan). The system was controlled by the Analyst 1.7.1 software.

### 2.10. Chromatography Conditions

Chromatography was performed under HILIC conditions using a SeQuant^®^ ZIC^®^-pHILIC 5 μm polymeric 150 mm × 2.1 mm PEEK-coated HPLC column (MilliporeSigma, Burlington, MA, USA). The column temperature, sample injection volume, and flow rate were set to 45 °C, 5 μL, and 0.15 mL/min, respectively. The HPLC conditions were as follows: Solvent A: 20 mM ammonium carbonate containing 0.1% ammonium hydroxide and 5 μM medronic acid. Solvent B: Acetonitrile. The gradient conditions were as follows: 0 min, 80% B; 20 min, 20% B; 20.5 min, 80% B; 34 min, 80% B. The total run time was 34 min. Data were processed using the SCIEX MultiQuant 3.0.3 software with relative quantification based on the peak area of each metabolite.

### 2.11. Metabolomics Data Analysis

Each metabolite in the sample was normalized as: C (normalized peak area) = peak area/P (protein amount in each sample). Metabolomics heat maps and PCA plots were generated using MetaboAnalyst 6.0, where the results were further normalized by sum and data were auto-scaled [(value − mean)/SD]. Box plots for nucleotide metabolites were generated using normalized concentration (C), and the *t*-test was run between latent and lytic samples as well as between different time points in latent and lytic samples. The significance is denoted as follows: *p*-value > 0.05 (ns); *p*-value > 0.01 (*); *p*-value > 0.001 (**); *p*-value < 0.001 (***).

### 2.12. qPCR for Intracellular KSHV Genomic DNA

After treatment with MTX, iSLK.BAC16 cells were pelleted at 300× *g* for 5 min at room temperature, the supernatant was removed, and the cells were harvested for intracellular KSHV viral genome quantification. DNA was extracted using the QIAamp DNA Blood Mini Kit (Qiagen, Venlo, The Netherlands, #51104) and stored at −80 °C until further use. qPCR was performed using PowerUp SYBR Green Master Mix (Applied Biosystems, Waltham, MA, USA #100029284) on a QuantStudio™ 3 Real-Time PCR System (Applied Biosystems, Waltham, MA, USA), with the LANA primers listed in Appendix A. The linearized LANA-HTBH plasmid was used to generate a standard curve (10–10 × e^10^ copies). After measuring the plasmid DNA concentration, copy number calculations were performed according to the following formula: copies/µL = [X g/µL DNA plasmid concentration/(plasmid length in base pairs × 660)] × 6.022 × 10^23^. Double-stranded beta-actin DNA standard (Origene, Rockville, MD, USA, HK200550), with known copies/µL, was used to generate a standard curve (10–10 × e^7^ copies). Standard curves (Ct vs. number of copies) were generated using the standards and used to calculate the copy number of each sample based on its Ct value. Viral intracellular copy number for LANA was normalized to actin copy number using the formula: normalized LANA _copy number_ = LANA _copy number_/Actin _copy number_. Data are presented as fold-change (FC) values: FC = normalized LANA _copy number (experimental)_/normalized LANA _copy number (control; +DOX+NaB)_. Experiments were conducted with a minimum of three independent biological replicates, and qPCR analysis was performed in triplicate for each sample. Statistical analysis was carried out using one-way ANOVA.

### 2.13. Statistical Analysis

Figures and statistical analyses were generated using the GraphPad Prism 8.0.1 software. Experiments were performed in at least three biological replicates. Data are presented as the mean ± SEM. KSHV experimental conditions were compared to control conditions using ANOVA. The MHV-68 viral titer was analyzed using a paired Student’s (two-tailed) *t*-test. Significant *p*-values are noted in each figure (* ≤ 0.05; ** ≤ 0.01; *** ≤ 0.001), and “ns” denotes a non-significant change.

## 3. Results

### 3.1. KSHV Lytic Infection Drives Global Metabolic Alterations

**Figure 1:**
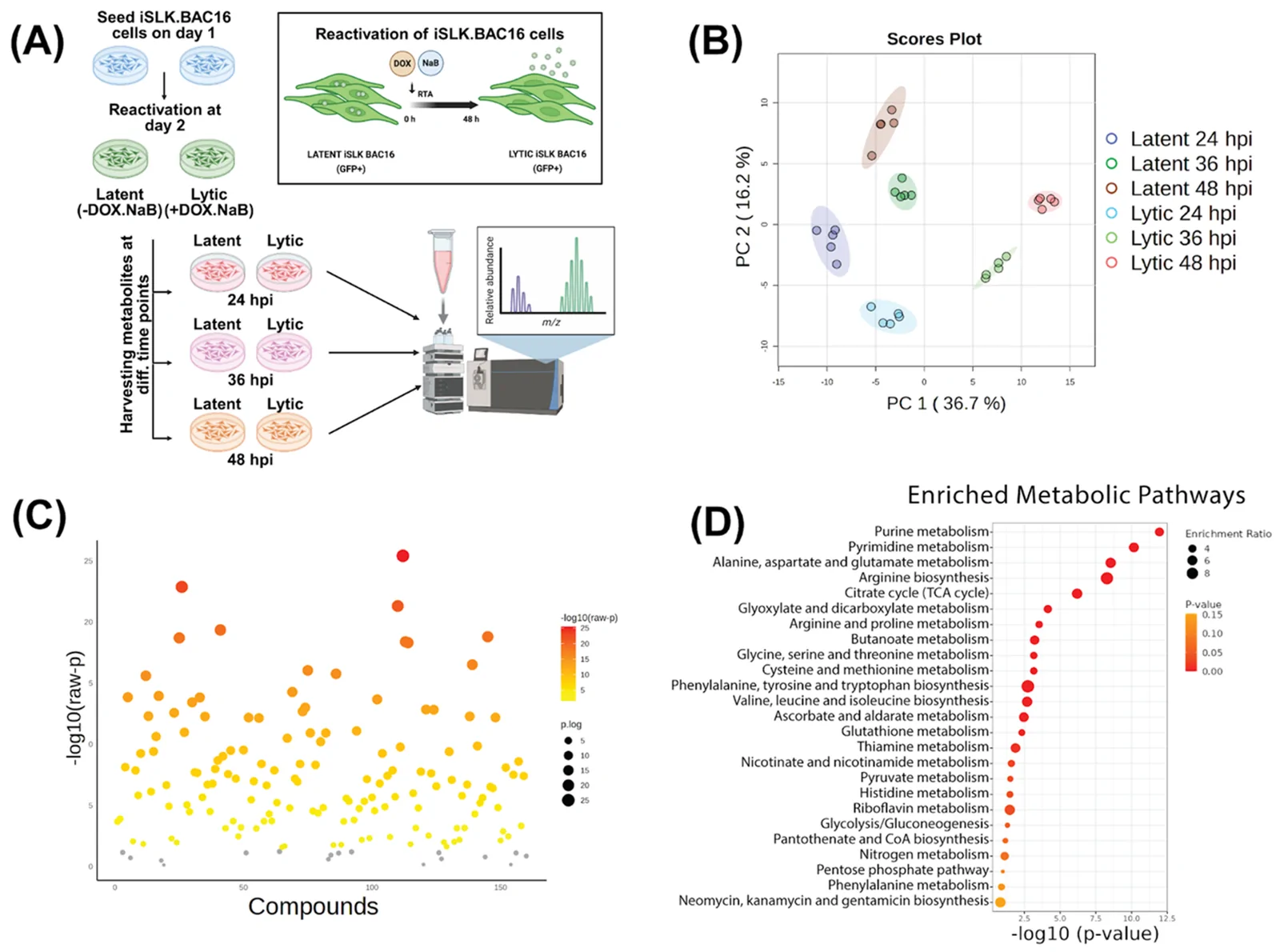
Targeted aqueous metabolomics of lytic vs. latent KSHV-infected iSLK.BAC16 cells. (**A**) Time-course experimental setup for targeted aqueous metabolomics of lytic vs. latent KSHV-infected iSLK.BAC16 cells using the LC-MS technique. (**B**) PCA depicting the separation of the samples. (**C**) Scatter plot illustrating the global pattern of significant metabolite changes across samples. Statistical significance was assessed using one-way ANOVA with Tukey’s HSD post hoc test. An FDR-adjusted *p*-value (q-value) < 0.05 was considered significant. Color intensity represents ANOVA *p*-values, with darker shades indicating greater statistical significance, while grey dots represent non-significant values (the full ANOVA statistics are provided in Supplemental Table S2). (**D**) Pathway enrichment analysis was performed on all the detected metabolites in the sample set, with purine and pyrimidine metabolic pathways being the most dysregulated. The size of the dots represents the enrichment ratio, which represents the number of metabolite hits for that pathway divided by the number of expected metabolite hits. The color of the dot represents the *p*-value, depicting the significance of that pathway being enriched. Significantly enriched pathways were identified using the “Pathway Enrichment Analysis” module based on the KEGG human reference library. Pathways are ranked by −log10(*p*-value), with larger values indicating greater statistical significance. Circle size reflects pathway impact based on topology analysis, and color indicates the degree of enrichment (from low to high).

iSLK.BAC16 cells stably maintaining the KSHV genome were used to determine the lytic viral metabolic profiles compared to latent infection at three different time points post Dox/NaB-mediated induction. Metabolomic analysis was performed at 24, 36, and 48 h post-induction (hpi) via Dox and NaB (Figure 1A). The targeted LC-MS analysis included five biological replicates normalized to protein concentration measured via BCA. A total of 160 metabolites were detected across the samples: 56 metabolites for amino acid metabolism, 61 metabolites for central carbon metabolism, and 44 metabolites for nucleotide metabolism (Supplemental Table S1). Principal Component Analysis (PCA) was conducted in MetaboAnalyst 6.0 to visualize global metabolic variation across samples. The score plot displaying each sample according to its principal components (PCs) revealed natural clustering patterns and differences in metabolic profiles between latent and lytic samples at different time points (Figure 1B). Separations along PC1 and PC2 indicate consistent metabolic shifts associated with the various experimental conditions, while overlaps indicate similarity between the sample sets (Figure 1B). One-way ANOVA was performed on the latent and lytic samples across different time points with a false discovery rate (FDR) cutoff of 0.05, and the data were subjected to Tukey’s Honestly Significant Difference (HSD) post hoc analysis. The scatter plot displays the significant differences in the abundances of metabolites across samples. Among the 160 detected metabolites, 143 metabolites exhibited statistically significant differences (Figure 1C, Supplemental Table S2). The *y*-axis corresponds to −log10(raw *p*-value), and the *x*-axis corresponds to the metabolites. The top discriminating metabolites include the highest FDR values, indicating strong between-group differences. Pathway enrichment analysis revealed the top two most significantly impacted and dysregulated pathways to be purine and pyrimidine metabolism (Figure 1D). Other pathways in the top 25 dysregulated pathways are amino acid metabolism, the citrate cycle, glutathione, arginine, and thiamine metabolism.

### 3.2. KSHV Lytic Infection Alters Amino Acid, Central Carbon, and Nucleotide Metabolism

**Figure 2:**
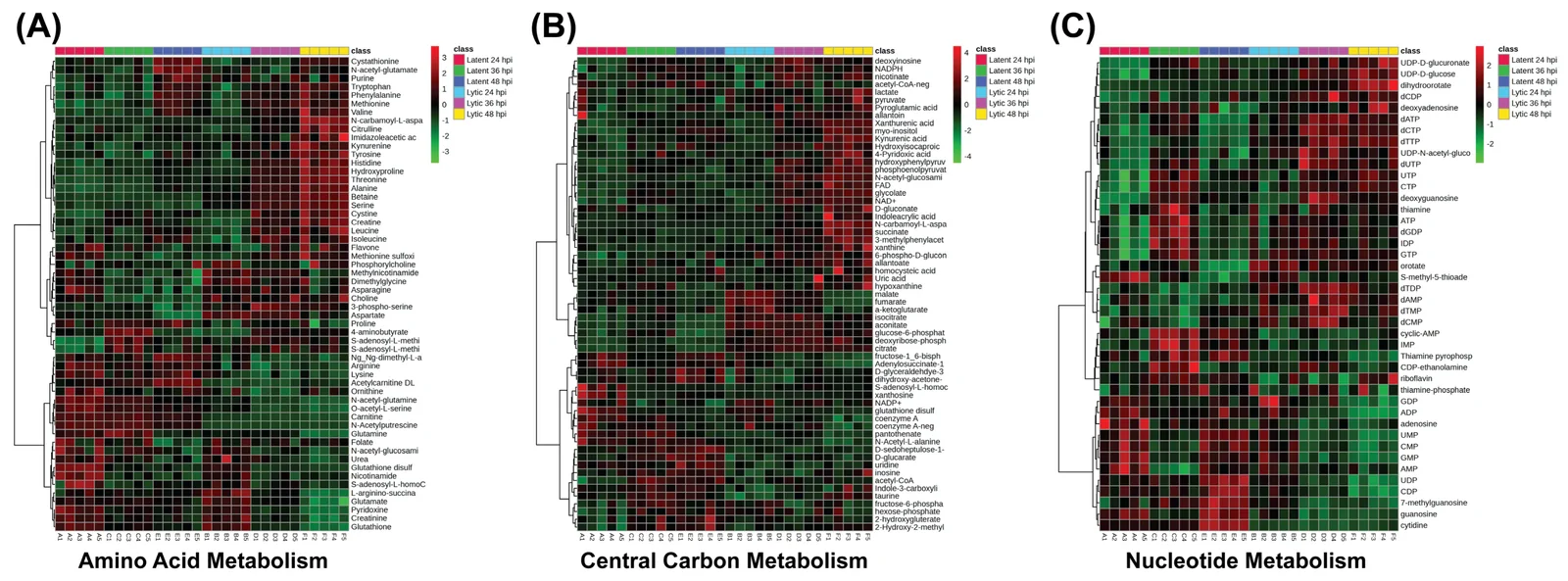
KSHV lytic infection alters key metabolic pathways. KSHV lytic infection induces metabolic alterations in host cells, including (**A**) amino acid, (**B**) carbohydrate, and (**C**) nucleotide metabolism. PCA plots show separation between samples. Metabolomics heat maps and PCA plots were generated using MetaboAnalyst 6.0, where the results were further normalized by sum and the data auto-scaled [(value − mean)/SD].

Upon deeper analysis of global metabolomics data from latent versus lytic KSHV infection, we found that the majority of metabolic pathways are altered during lytic infection. KSHV lytic infection alters amino acid metabolism, glycolysis, lactate production, glutaminolysis, fatty acid metabolism, and nucleotide metabolism, with increased amino acid monomers during lytic infection at different hours post-induction (hpi), i.e., 24, 36, and 48 hpi (Figure 2A, Supplemental Figure S1). At 48 hpi, we measured an increase in amino acid monomers in lytic samples compared to latent samples. The essential amino acids histidine, isoleucine, leucine, methionine, phenylalanine, threonine, tryptophan, and valine have higher abundances in lytic samples at 36 and 48 h post-induction. The metabolites associated with central carbon pathways were highly abundant in lytic KSHV samples when compared to latent (Figure 2B, Supplemental Figure S1). Additionally, we measured an increase in glycolytic metabolites, including glucose-6-phosphate (G6P), phosphoenolpyruvate (PEP), and pyruvate, and energy-related metabolites like NAD^+^, NADPH, and FAD during KSHV lytic infection (Figure 2B, Supplemental Figure S1). In lytic KSHV samples, we also measured an increase in TCA metabolites, mostly at 24 and 36 hpi, which are involved in reactions producing energy-rich molecules (NAD^+^ and FAD), such as isocitrate, alpha-ketoglutarate, fumarate, and malate. Furthermore, metabolites of nucleotide metabolism demonstrated differential abundance across the time course (Figure 2C). At both 24 hpi and 48 hpi, we observed a trend toward increased levels of nucleotide metabolites and precursors of DNA and RNA: dCTP, dATP, dTTP, UTP, CTP, ATP, and GTP. The nucleotide metabolites showed a cumulative increase at both 24 and 48 hpi, suggesting an increased nucleotide metabolism during lytic infection. The UDP sugar pool essential for gluconeogenesis and the glycosylation of proteins (UDP-glucose, UDP-glucuronate, and UDP-N-acetylglucosamine) was increased at 36 and 48 hpi during lytic infection [33] (Figure 2C).

### 3.3. Lytic KSHV Infection Increases DNA and RNA Nucleotide Biosynthesis

**Figure 3:**
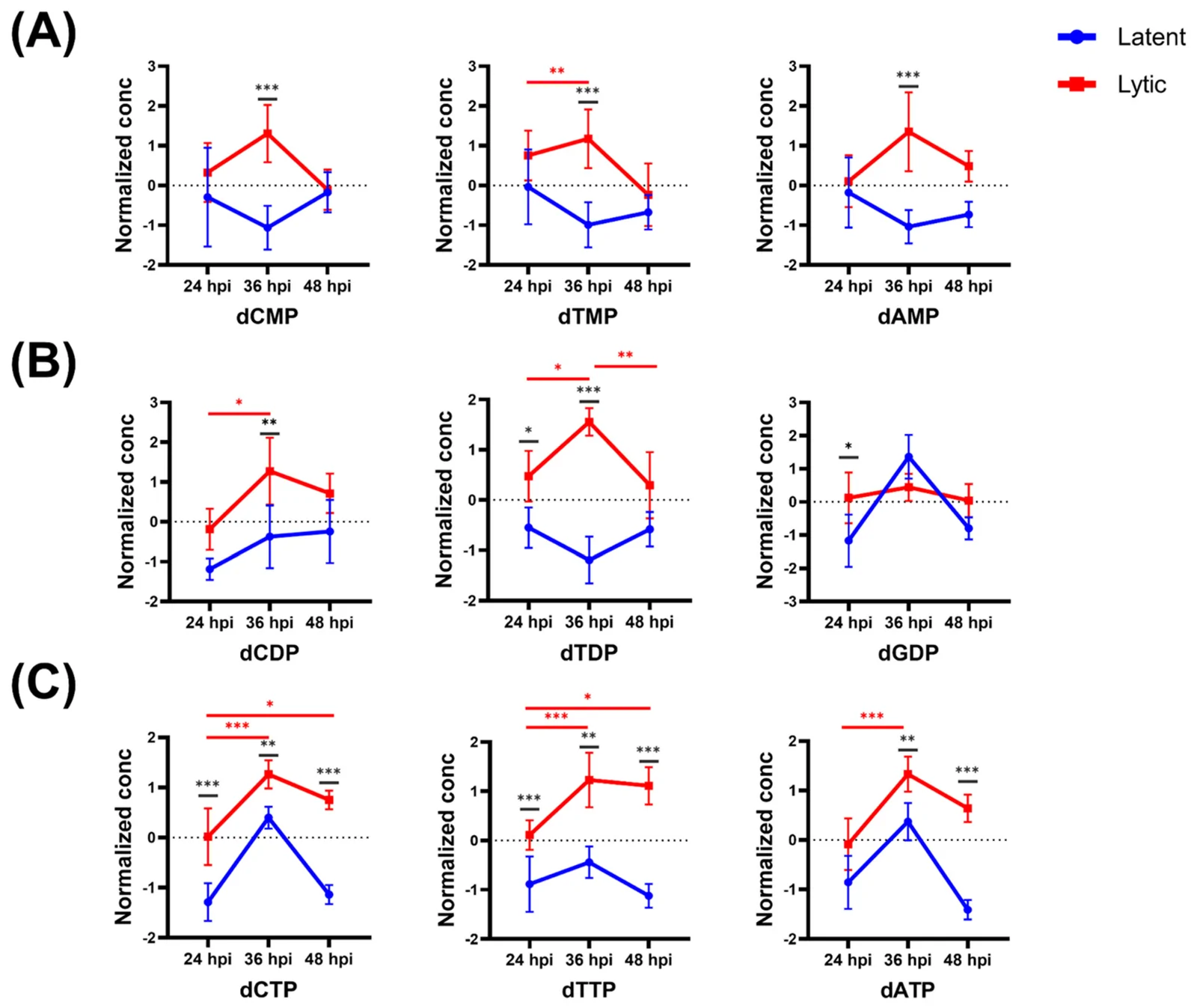
Deoxynucleotide metabolites are altered during lytic KSHV infection for maximal viral replication. iSLK.BAC16 cells, treated with or without Dox/NaB for reactivation, were harvested at 24, 36, and 48 h post-induction (hpi). Deoxynucleotide phosphate species in (**A**) (dCMP, dTMP, and dAMP) and (**B**) (dCDP, dTDP, and dGDP) were significantly abundant at 36 hpi when compared to latent infection, while those in (**C**) (dCTP, dTTP, and dATP) were significantly elevated at both 36 and 48 hpi when compared to latent infection. Red lines represent lytic KSHV-infected samples, and blue lines represent latent KSHV infection. ***, *p* ≤ 0.001; **, *p* ≤ 0.01; * ≤ 0.05; *n* = 5.

Deoxynucleotide triphosphates (dNTPs) are used to synthesize DNA (DNA replication), while nucleotide triphosphates (NTPs) are used in RNA synthesis (transcription) [34]. DNA and RNA, being polynucleotide chains, are composed of purines (adenine and guanine) and pyrimidines (thymidine, cytidine, and uridine). In our LC-MS data, we report that mono- and di-phosphate deoxynucleotides (dNMPs and dNDPs) show a significant increase at 36 hpi, but their levels are reduced at 48 hpi compared to latent samples (Figure 3A,B). However, the dNTPs (dATP, dCTP, and dTTP) showed significant elevation at 36 hpi, and their levels remained elevated through 48 hpi when compared to earlier time points (Figure 3C). Interestingly, dNTPs remained elevated at 48 hpi, which could be due to the conversion of dNMPs and dNDPs into dNTPs to fulfill the need of the high DNA replication mechanism during lytic KSHV infection (Figure 3C).

**Figure 4:**
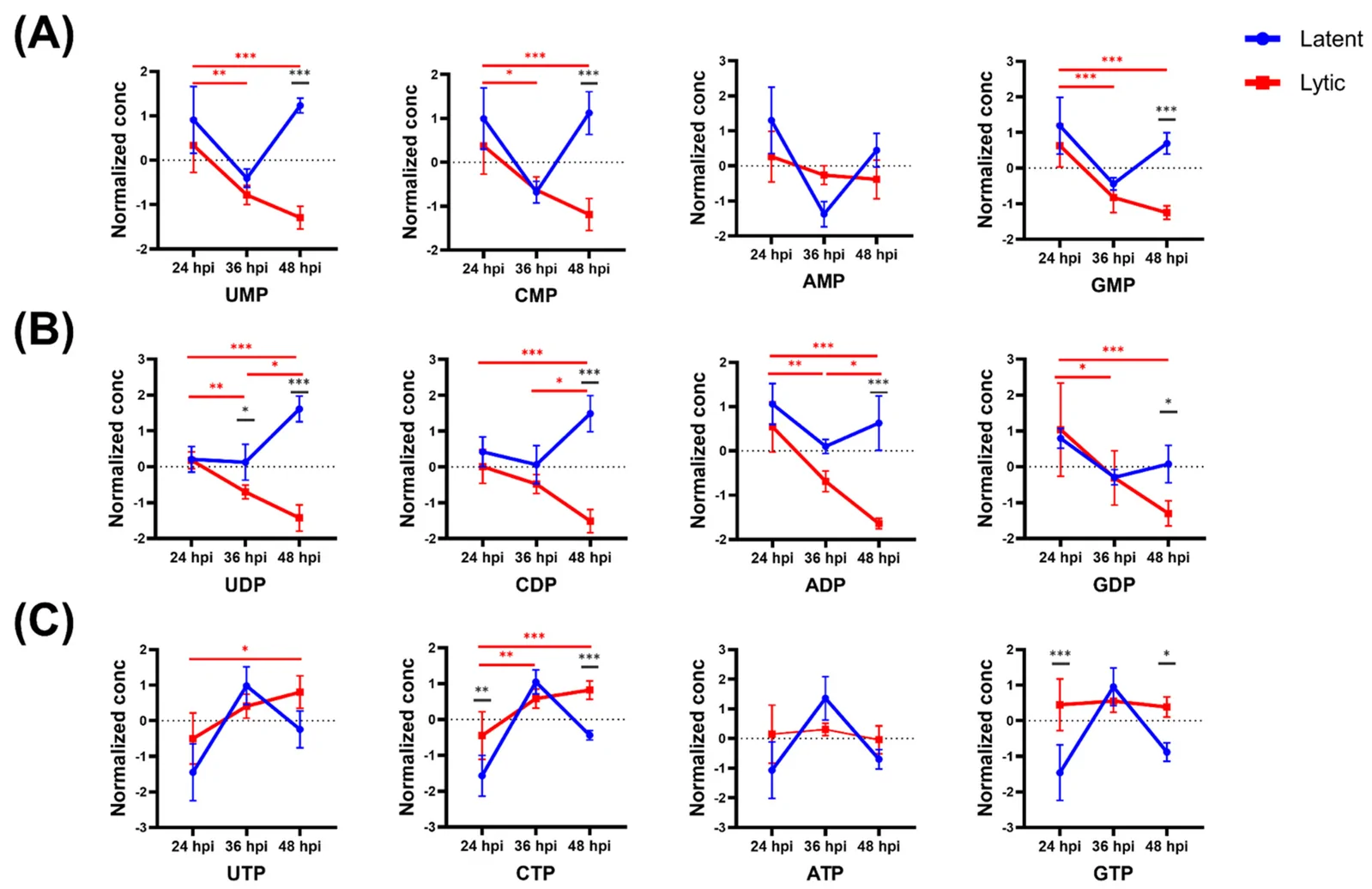
Nucleotide metabolites are altered during lytic KSHV infection for maximal virion production. iLSK.BAC16 cells, treated with or without Dox/NaB, were harvested at 24, 36, and 48 h post-infection. Nucleotide phosphate species—(**A**) UMP, CMP, AMP, and GMP; (**B**) UDP, CDP, ADP, and GDP; (**C**) UTP, CTP, ATP, and GTP—were significantly elevated at 48 hpi compared to latent infection. Red lines represent lytic KSHV-infected samples, and blue lines represent latent KSHV infection. ***, *p* ≤ 0.001; **, *p* ≤ 0.01; *, *p* ≤ 0.05; *n* = 5.

Next, we see that mono- and di-nucleotide phosphates (NMPs and NDPs) are significantly lower at 36 and 48 hpi compared with 24 hpi (Figure 4A,B). Triphosphate pyrimidines (UTP and CTP) and purines (ATP and GTP) are incorporated during RNA production. NTPs, especially pyrimidines, were significantly elevated during lytic infection at 48 hpi when compared to 24 hpi, as shown in the box plots (Figure 4C). In comparison, at 48 hpi, during lytic infection of iSLK.BAC16 cells, NMPs (CMP and CDP) (Figure 4A) and NDPs (CDP and GDP) (Figure 4B) are significantly low in abundance, and NTPs (CTP and GTP) (Figure 4C) are significantly high when compared to latent infection. This elevation in NTPs suggests that KSHV requires active nucleotide metabolites (triphosphate species) for high rates of DNA transcription and RNA production at 48 hpi.

Apart from being the key unit of RNA polymer, ATP is the vital energy currency of the cells for all biosynthetic pathways [35]. GTP is converted to ATP during times when high cellular energy is required. The box plots of ATP and GTP show an elevation at all time points (24, 36, and 48 hpi) during lytic infection when compared to latent infection (Figure 4C).

### 3.4. Nucleotide Metabolism Is Required for Maximal Virion Production During Lytic Gammaherpesvirus Infection

**Figure 5:**
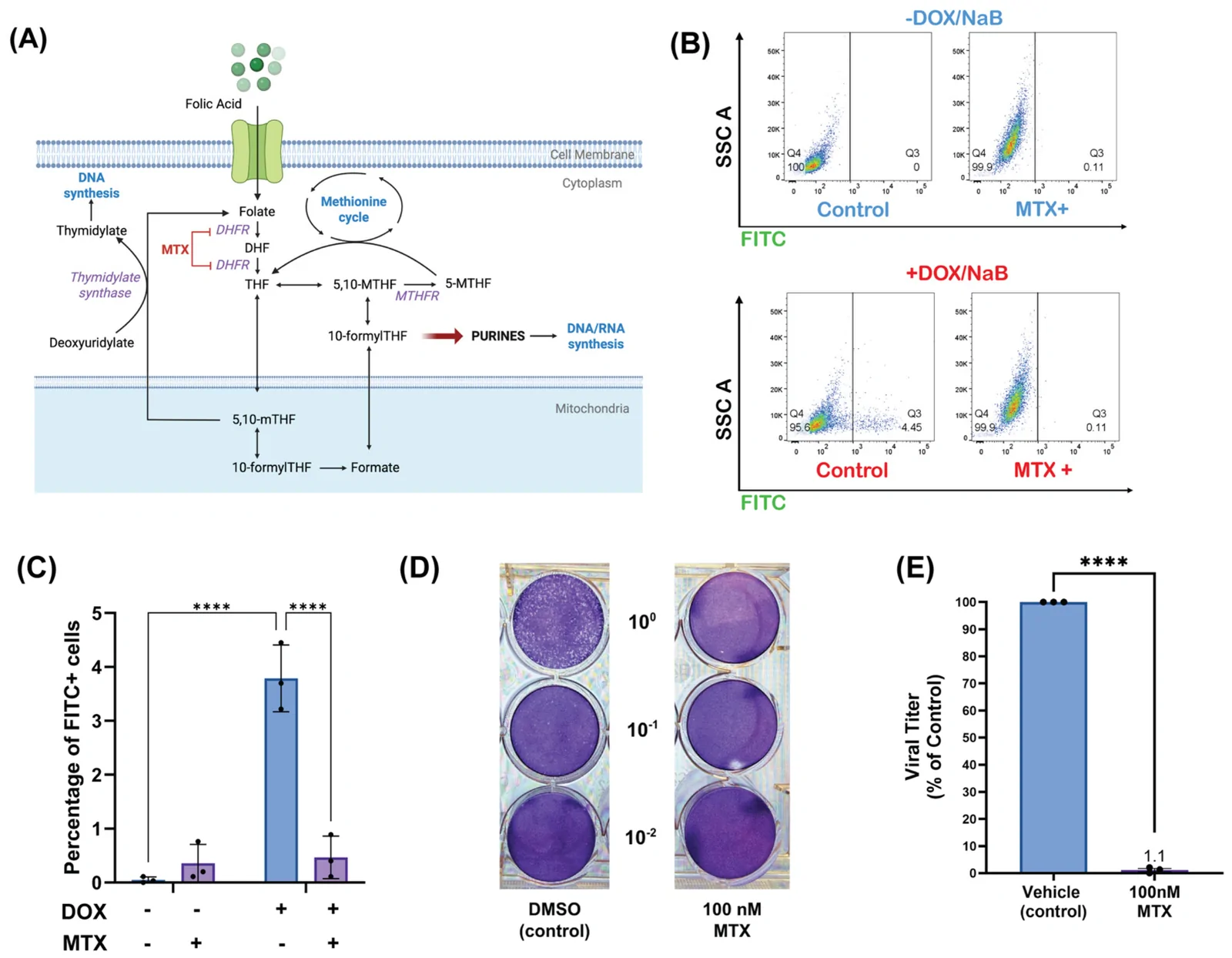
Methotrexate inhibition of nucleotide metabolism decreases infectious KSHV production. (**A**) Mechanism of action of methotrexate (MTX) during folate metabolism in the host cell. (**B**) Representative flow plots of GFP expression/FITC+ signal in iSLK.BAC16 cells. (**C**) iSLK.BAC16 cells were reactivated with 1 μg/mL Dox and 1 mM NaB for 48 h and treated with methotrexate simultaneously. Flow data were analyzed with latent samples as controls. The percentage of FITC+ cells decreased from ~4% in the control to 0.4% in MTX-treated lytic cells. (**D**,**E**) NIH 3T3 cells were MHV-68 infected (MOI = 0.1) and treated with vehicle (DMSO control) or 100 nM MTX for 48 h in three independent experiments. Viral titer was quantified from extracellular supernatants via plaque assays. (**D**) Representative MHV-68 plaque assay images from vehicle (control) or 100 nM MTX-treated cells from 10-fold dilutions. (**E**) Quantification of MHV-68 extracellular viral titer as a percentage of the control (vehicle treatment). ****, *p* ≤ 0.0001; *n* = 3.

To understand the requirement of nucleotide metabolism during KSHV lytic infection, we used an FDA-approved methotrexate (MTX), an antimetabolite drug. MTX, a folate antagonist, competitively inhibits the host enzyme dihydrofolate reductase (DHFR). DHFR is required for the conversion of folic acid to its active forms, dihydrofolate (DHF, FH2) and tetrahydrofolate (THF, FH4). MTX treatment creates depletion of THF cofactors, including 5-methyl-THF, and thereby blocks several folate-related metabolic processes, including inhibition of nucleotide metabolism (Figure 5A). THF cofactor depletion in the host cells affects the de novo synthesis of pyrimidine, deoxythymidine, required for DNA synthesis and the de novo synthesis of purine, guanosine, and adenosine, required for both DNA replication and transcription [36].

To test if elevated nucleotide metabolism is required for infectious KSHV virion production, we treated iSLK.BAC16 cells with 125 nM MTX, which displayed minimal cytotoxicity (Supplemental Figure S2). Cell-free supernatants were collected after 48 hpi and titered on iSLK cells to determine viral production. The infected iSLK cells were washed twice with PBS and trypsinized, and the cells were resuspended in FACS buffer. After fixing, the samples were analyzed for FITC expression by flow cytometry. For each sample, 10,000 cellular events were recorded. In the control samples treated with only DOX and Nab, ~4% of cells expressed FITC/GFP (Figure 5B). However, in lytic samples treated with MTX, there was a significant decrease in FITC-expressing cells, suggesting that there was a significant decrease in viral load after MTX treatment during lytic infection (Figure 5C). Moreover, while there was approximately a 15% reduction in cell viability, we observed about a 97.5% reduction in virus production (Supplemental Figure S2E). These data confirm that nucleotide metabolism is required for infectious KSHV virion production.

As previously noted, metabolomics analysis revealed that lytic MHV-68 infection increases nucleotide metabolism [25]. To test if MTX treatment also reduced lytic infectious virus production during mouse gammaherpesvirus infection, we mock- or MHV-68-infected (MOI = 0.1) NIH 3T3 cells and then treated them with DMSO (vehicle control) or 100 nM MTX for 48 h. While 100 nM MTX treatment reduced cell proliferation compared to vehicle (control), 100 nM MTX treatment resulted in 99% cell viability in mock- and MHV-68-infected NIH 3T3 cells (Supplemental Table S3). Quantitative plaque assay analysis revealed that 100 nM MTX treatment of MHV-68-infected cells decreased infectious virus production by 98.9% compared to control-treated cells (Figure 5D,E). After normalization of the data for the viral titer to live cell number, our data showed a ~98.5-fold decrease in virus production compared to control-treated cells. Overall, these data suggest that the induction of nucleotide metabolism by both human (KSHV) and mouse (MHV-68) gammaherpesviruses is necessary for virion production.

### 3.5. Nucleotide Metabolism Is Required for KSHV Viral Copy Number and Late Lytic KSHV Gene Expression

**Figure 6:**
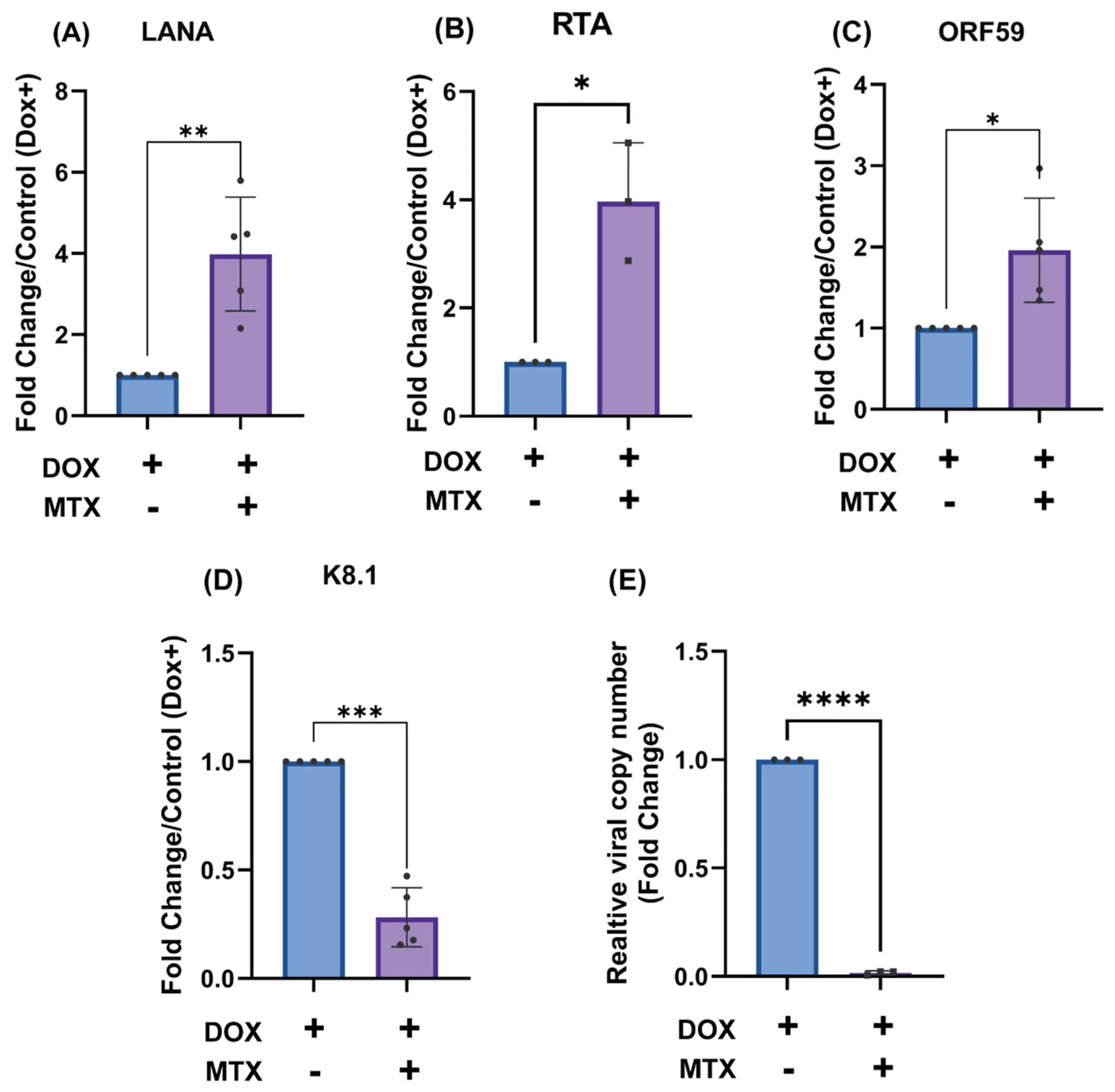
Methotrexate inhibition of nucleotide metabolism decreases intracellular KSHV copy number and late lytic gene expression. iSLK.BAC16 cells were induced in the presence or absence of 125 nM MTX. Cells were harvested at 48 hpi. RNA was extracted, and qRT-PCR was performed on cDNA for (**A**) latent KSHV transcript (LANA), (**B**,**C**) early KSHV transcripts (RTA and ORF59), and (**D**) late KSHV transcript (K8.1). Relative mRNA expression for each viral transcript was normalized to a housekeeping gene and compared to that of untreated induced samples. (**E**) Intracellular viral copy number was calculated upon MTX treatment during lytic reactivation. Error bars are representative of standard errors from five biological replicate experiments. ****, *p* ≤ 0.0001; ***, *p* ≤ 0.001; **, *p* ≤ 0.01; *, *p* ≤ 0.05; *n* = 3 or 5.

To determine how MTX affects lytic infection, the iSLK.BAC16 cells were treated with and without 125 nM MTX, which displayed minimal cytotoxicity in the presence or absence of Dox/NaB to induce the lytic phase (Supplemental Figure S2). The cells were reactivated at the same time as MTX treatment to make sure nucleotide synthesis was inhibited, and there was no further de novo production of nucleotides during reactivation. After 48 hpi, the supernatant was collected and stored for titer experiments. RNA was extracted from cells, and viral gene expression was quantified using RT-qPCR. The relative mRNA expression level of the latent gene LANA showed a significant increase after MTX treatment (Figure 6A, Supplemental Figure S3). Next, we analyzed the mRNA expressions of the early lytic genes, RTA and ORF59, and a late lytic gene, K8.1. RTA and ORF59 mRNA expressions significantly increased after MTX treatment (Figure 6B,C, Supplemental Figure S3). Interestingly, we measured a significant decrease of about 70% in K8.1 relative mRNA levels (Figure 6D).

Next, we determined the KSHV genome copy number after MTX treatment during lytic replication to understand at which step of the viral replication cycle the inhibition of nucleotide metabolism affected lytic viral infection. The intracellular viral genome copy number was evaluated by qPCR, using primers that recognize the LANA coding sequence with and without MTX treatment during reactivation (Figure 6E). Our data demonstrated a 99-fold decrease in the KSHV viral genome compared to the reactivated control. These findings suggest that host nucleotide metabolism is required for KSHV lytic infection, specifically at the viral genome replication stage. Furthermore, drug inhibition of nucleotide metabolism shows that KSHV lytic infection is dependent on the host metabolic pathways in order to produce infectious KSHV virions.

## 4. Discussion

In this study, we demonstrate that nucleotide metabolism is elevated and required by KSHV lytic infection for maximal virion production and thus represents a potential therapeutic target for treating KSHV infection. Alterations in the global host metabolome have been previously studied in the presence of other viral infections such as HCMV, vaccinia virus (VACV), HCV, EBV, and HSV-1, as well as KSHV latent infection [13,16,17,18,19,22,37]. Our PCA shows a clear separation between latent and lytic samples, indicating a unique metabolic profile of KSHV infection at 24, 36, and 48 hpi in both viral phases (Figure 1B). In this paper, we have defined the altered metabolic pathways in KSHV lytic infection compared to latent infection at various time points post-lytic induction (Figure 1C). Using LC-MS targeted metabolomics on KSHV latent and KSHV lytic samples collected at several time points, we were able to perform data analysis and pathway enrichment analysis to determine significantly altered metabolic pathways (Figure 1D). We found that the pathway most altered in KSHV lytic samples was nucleotide metabolism. Other top pathways altered during KSHV lytic infection included amino acid metabolism, the citrate cycle, glutathione metabolism, arginine metabolism, and thiamine metabolism. Glutathione metabolism is required to maintain the redox homeostasis of the host cell. KSHV-encoded microRNAs regulate the expression of the xCT transporter (SLC7A11) and protect the cells from reactive nitrogen species (RNS) to protect the cells from extreme redox environments and help KSHV persist in infection [38]. Another study confirms that the KSHV ORF 59 lytic gene modulates histone H4 arginine methylation, which controls the compactness of the chromatin and induces lytic reactivation, thus altering the arginine metabolic pathway [39].

In our LC-MS dataset, we see an increase in anabolic metabolites during KSHV lytic infection. An increase in the abundance of essential amino acids—histidine, isoleucine, leucine, methionine, phenylalanine, threonine, tryptophan, and valine—suggested the demand for protein synthesis to fulfill viral replication needs (Figure 2A). A study performed on HSV-1 demonstrated the requirement of essential amino acids for higher viral load [40]. Among the essential amino acids in the LC-MS data, only lysine was decreased in lytic KSHV infection. Lysine residues are the major sites for post-translational modifications (PTMs) of both viral and host proteins, which KSHV manipulates to dysregulate PTMs and evade the host immune response [41,42,43]. Interestingly, we see higher glutamine presence at 24 and 36 hpi in latent samples. Previous groups have established that KSHV latent infection induces glutaminolysis and enhances glutamine uptake [44,45] (Figure 2A). Further analysis of the upregulated metabolic pathways showed an increase in the energy-related metabolites of NAD^+^, NADPH, and FAD during lytic KSHV infection and an increase in the metabolites that are involved in reactions with these energy metabolites, such as glucose-6-phosphate (G6P), phosphoenolpyruvate (PEP), and pyruvate in glycolysis and isocitrate, alpha-ketoglutarate, fumarate, and malate in the TCA cycle (Figure 2B). Furthermore, an increase in lactate abundance implies that there is a Warburg effect occurring in KSHV lytic samples. The Warburg effect is defined as the cellular usage of glycolysis for energy production instead of oxidative phosphorylation, even in the presence of oxygen, in order to obtain rapid energy production and ensure viral replication [20]. The role of the Warburg effect has been established in latent KSHV infection in previous studies [20]. The increase in nucleotide-related metabolites at both 24 and 48 hpi suggests an increased requirement for nucleotide metabolism during KSHV lytic infection (Figure 2C). Since the KSHV replication cycle includes two phases of DNA replication—immediate-early and early lytic gene replication, followed by genome replication—we hypothesize that an increase in nucleotides at 24 hpi compensates for early gene expression. However, a more pronounced difference in nucleotides at 48 hpi is due to genome replication. Since genome replication is also followed by transcription and translation of viral proteins, this further suggests that the increase in amino acids at 48 hpi is for viral protein formation (Figure 2C). Our LC-MS data show significantly increased abundances of these UDP-sugars at 36 and 48 hpi during lytic KSHV infection (Figure 2C). All herpesviruses, including KSHV, have glycoproteins on the outermost lipid membrane for initiating binding of host cell receptors either during new infection or egress from the host cell [46,47,48]. UDP sugars such as UDP-glucose, UDP-glucuronate, and UDP-N-acetylglucosamine serve as glycosylation substrates during post-translational modifications [49]. KSHV encodes eight glycoproteins, which are incorporated into the virion envelope: ORF8 (gB [glycoprotein B]), ORF28, ORF68, ORF22 (gH), ORF47 (gL), ORF39 (gM), ORF53 (gN), and gpK8.1 [50,51]. These glycoprotein structures share homology with those of other herpesviruses or are unique to KSHV itself [52,53,54].

Finally, our data show that deoxynucleotides are significantly higher in abundance at 36 and 48 hpi during lytic infection compared to latent samples, suggesting that DNA replication occurs throughout lytic KSHV infection (Figure 3). Upon reactivation, an organized cascade of gene expression is initiated with immediate-early and early lytic gene expression, viral genome replication, and late lytic gene expression [55]. The host metabolomic machinery aids KSHV in viral DNA replication and gene expression [56]. However, the increase in nucleotide phosphate species at 48 hpi suggests that more genome transcription is required during the later phase of the viral lytic replication cycle (Figure 4). We determined the requirement for nucleotide metabolism during lytic infection by demonstrating that inhibiting de novo nucleotide production indirectly with the chemical MTX decreased intracellular KSHV viral copy number and reduced late lytic viral gene expression, K8.1, as well as extracellular virion production (Figure 5 and Figure 6). MTX, a folate antagonist, binds tightly (competitively) and inhibits the host enzyme dihydrofolate reductase (DHFR). DHFR is a key enzyme in folate metabolism, which recycles folic acid to THF [57]. MTX treatment creates depletion of THF cofactors and thereby blocks several folate-related metabolic processes, one of which is inhibition of nucleotide metabolism [58]. MTX has a higher affinity for DHFR than folic acid and hence is used at low cellular doses to decrease death by toxicity [30,59]. Notably, lytic KSHV encodes a viral DHFR (ORF2), which is catalytically more active and does not show inhibition by known inhibitors of the DHFR enzyme, including MTX [58,60].

Additionally, as reported for other herpesviruses, KSHV relies on the host cell for nucleotide metabolism [16,18,54,61]. However, the KSHV genome encodes various enzymes involved in nucleotide synthesis, including thymidine kinase (TK, ORF21), thymidylate synthase (TS, ORF70), and dihydrofolate reductase (DHFR, ORF2), which may induce host metabolic reprogramming during lytic infection [60]. Among these, TK is a key enzyme in the salvage pathway that recycles thymidine. During lytic KSHV infection, since MTX only inhibits de novo nucleotide synthesis, nucleotides can be recycled via the salvage pathway. In our data on iSLK.BAC16 cells, we see an increased expression of the immediate-early gene, RTA, and early lytic gene, ORF59, followed by a significant decrease in viral copy number and the late lytic viral gene, K8.1. Whether KSHV specifically upregulates the salvage pathway after MTX treatment has not been clearly established in the field. It is important to note that the salvage pathway usually cannot completely replace de novo synthesis during periods of very high nucleotide demand, such as during KSHV lytic replication. In this case, the cells would have to rely on de novo nucleotide synthesis despite the higher energy requirements. Hence, an increase in early lytic gene expression may be a result of the activation of the salvage pathway. Nevertheless, de novo nucleotide synthesis is still an essential requirement for the progression of lytic KSHV infection, and its inhibition demonstrates a significant decrease in infectious virion production.

**Figure 7:**
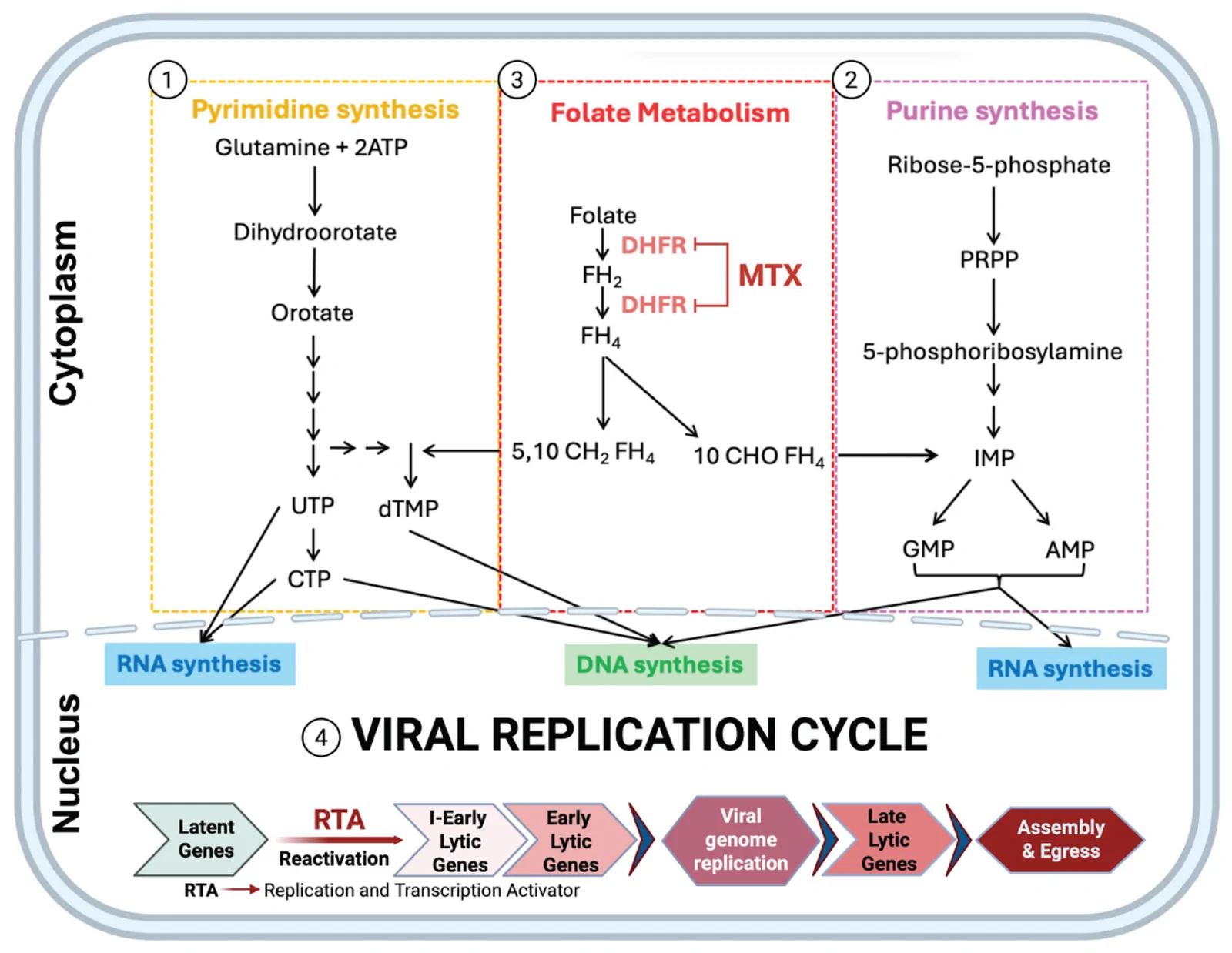
Summary model of host cell nucleotide metabolism changes during lytic gammaherpesvirus infection. Our time-course global metabolomics indicates that during KSHV lytic infection of iSLK.BAC16 cells, metabolic intermediates found in nucleotide metabolism are significantly elevated in lytic samples when compared to the latent samples. In the host cell, de novo nucleotide synthesis includes pyrimidine and purine synthesis, which occurs in the host cytoplasm. This figure illustrates how de novo pyrimidine and purine biosynthesis pathways support viral replication following reactivation from latency. (1, 2) Pyrimidine synthesis (left) generates UTP, CTP, and dTMP, while purine synthesis (right) produces IMP-derived GMP and AMP; both pathways converge on nucleotide pools required for viral RNA and DNA synthesis. (3) Central folate metabolism (red box), which supplies one-carbon units for thymidylate and purine production, is shown as a key regulatory node catalyzed by dihydrofolate reductase (DHFR) and inhibited by methotrexate (MTX). (4) In the nucleus (bottom), the viral replication cycle is depicted, beginning with RTA-mediated reactivation, followed by immediate-early, early, and late lytic gene expression, viral genome replication, and assembly/egress. Together, the schematic highlights that robust nucleotide biosynthesis is essential for efficient herpesvirus lytic replication.

In conclusion, our findings define and expand our understanding of the host metabolic profile during lytic gammaherpesvirus infection (Figure 7). We demonstrate a unique metabolic profile of host cells undergoing lytic KSHV infection at 24, 36, and 48 h post-induction. Gammaherpesviruses, including KSHV, MHV-68, and EBV, exploit host nucleotide metabolism to establish persistent viral infection and replication, leading to a diseased state [24,25,62,63,64]. Reduction in viral titers by MTX-mediated nucleotide metabolic inhibition shows that there is a shared metabolic vulnerability among gammaherpesviruses. The findings of this study provide a mechanistic insight into how gammaherpesviruses exploit host metabolic pathways to sustain lytic replication.

## Data Availability

Data is contained within the article or Supplementary Materials.

## Supplementary Materials

The following supporting information can be downloaded at https://www.mdpi.com/article/10.3390/v18080877/s1. Supplemental Figure S1: Metabolic alterations in host cells during KSHV lytic infection. Supplemental Figure S2: Methotrexate exhibits minimal cytotoxic effects at 125 nM. Supplemental Figure S3: KSHV viral gene expression in iSLK.BAC16 cells. Supplemental Table S1: Time-course metabolomics data normalized to protein concentration. Supplemental Table S2: ANOVA statistics. Supplemental Table S3: Methotrexate exhibits minimal cytotoxic effects at 100 nM in mock- and MHV-68-infected NIH3T3 cells.

## Abbreviations

The following abbreviations are used in this manuscript:

KSHV	Kaposi’s Sarcoma Herpes Virus
KS	Kaposi’s Sarcoma
LANA	Latency-Associated Nuclear Antigen
ORF59	Viral DNA Polymerase
K8.1	Viral Glycoprotein
LC-MS	Liquid Chromatography–Mass Spectrometry
GC-MS	Gas Chromatography–Mass Spectrometry
MHV-68	Murine Herpesvirus 68
MTX	Methotrexate
hpi	Hours Post Induction

## Appendix A. Primers

**Target**	**Model**	**Forward Primer**	**Reverse Primer**	**Application**
LANA	KSHV	GTGACCTTGGCGATGACCTA	CAGGAGATGGAGAATGAGTA	RT-qPCR
RTA	KSHV	CAA GGT GTG CCG TGT AGA GAT	GGT CAA AGC CTT ACG CTT CTT	RT-qPCR
ORF59	KSHV	GACAGCGTCTCGCTGACAGA	CACACGCGTGAGCTATTCGG	RT-qPCR
K8.1	KSHV	TAACCATGATTCCACACAGATTC	GGTTTTGTGTTACACTATGTAGG	RT-qPCR
Actin	Human	CACCATTGGCAATGAGCGGTTC	AGGTCTTTGCGGATGTCCACGT	RT-qPCR
LANA	KSHV	GGAACGCGCCTCATACGA	CGCGAATACCGCTATGTACTCA	qPCR
